# Supplementary material for: Treatment profiles and clinical outcomes of COVID-19 patients at private hospital in Jakarta
Source: PLoS One. 2021 Apr 16;16(4):e0250147. doi: 10.1371/journal.pone.0250147 (PMC8051784; doi:10.1371/journal.pone.0250147)
Supplement: Attachment — Submitted filename: Response to Reviewers.docx [file pone.0250147.s002.docx]

REBUTAL LETTER

FOR EDITOR

1. We have improved the article according to the guidelines
2. We have corrected the significant text overlap (**all the read colour)**
3. We have provided the details regarding participant consent (in Methodology). Actually, my student, Suri Isnaini, worked as a part-time job in the hospital's pharmacy department. That’s why she could get the data because she was one of the staff who prepared the medication for the patients.
4. We have corrected the spelling and the grammar from the English center of our Univ

For Reviewer

1. Is the manuscript technically sound, and do the data support the conclusions?

We have corrected the conclusion

2. Has the statistical analysis been performed appropriately and rigorously?

Yes, we just used Chi-Square and Kaplan Meier. It based on our data only

3. Reviewer #1: The authors aimed to find out the effective treatment as an antiviral agent for COVID-19, to determine the correlation between sociodemography with clinical outcomes and duration of treatment, and to determine the relationship between comorbidities with clinical outcomes and duration of treatment for COVID-19 patients.
   The study, overall considered, is very interesting for the journal but in the discussion I would deepen a little more the issue of concomitant cardiovascular disease and outcomes. For example cite and discuss the paper by Ielapi N et al. Cardiovascular disease as a biomarker for an increased risk of COVID-19 infection and related poor prognosis. Biomark Med. 2020;14(9):713-716.

(**we have added as a reviewer suggestion in the yellow colour**)

Reviewer #2: This is the first submission for an article titled “Treatment profiles and clinical outcomes of COVID-19 patients at private hospital in Jakarta”. The project has a well written introduction. I am largely concerned about the manuscript sample size and the number of analyses completed with the size of the sample. The statistical plan is also not fully articulated relative to the aims. Additionally, the style of the paper is not traditional and would need to be altered to the type of the journal and typically accepted format.

(We have did correction for the format and style of manuscript according to the guideline, and for the analysis we just do Chi-square and Kapplan Meier) the revision of methodology appears **in yellow**.

**We did paraphrasing to reduce the similarity in Blue colour**
